# Identification of *Fasciola hepatica* gut-associated glycoproteins as potential vaccine candidates by lectin-affinity chromatography, flukicidal monoclonal antibodies, and affinity-enrichment mass spectrometry

**DOI:** 10.3389/fpara.2026.1746419

**Published:** 2026-05-29

**Authors:** Sonja G. Rechsteiner, Sina Hasler, Peter Gehrig, Peter Deplazes, Ramon M. Eichenberger

**Affiliations:** 1 Institute of Parasitology, Vetsuisse Faculty, University of Zurich, Zurich, Switzerland; 2 Functional Genomics Centre Zurich, Swiss Federal Institute of Technology Zurich (ETH Zurich), University of Zurich, Zurich, Switzerland; 3 Medical Micro- and Molecular Biology, Institute of Chemistry and Biotechnology, Zurich University of Applied Sciences (ZHAW), Wädenswil, Switzerland

**Keywords:** *Fasciola hepatica*, gut protein, LFQ-proteomics, liver fluke, mass spectrometry, monoclonal antibody, pull-down, vaccine

## Abstract

A significant economic impact to the agriculture and declining drug efficacy due to anthelmintic resistance lead to the search for novel intervention approaches against the zoonotic liver fluke *Fasciola hepatica*. A cost-effective strategy is to induce protective immunity by vaccination. However, adaptation of the parasite to evade the host immune response hampers classical vaccine development. Therefore, an ideal protective epitope would not be subjected to this evolutionary selection. Antigens, which are not exposed to the host’s immune system during a natural parasite infection, but which could be attacked by vaccine antibodies, have been shown to be valid candidates, especially those expressed in the gut of blood-feeding and tissue-dwelling helminths. Overall, helminth gut-associated glycoproteins—including hidden antigens—serve as a rich source for novel anthelminthic vaccine candidates. Here, we purified *F. hepatica* gut-associated glycoproteins assisted by lectin-affinity chromatography and specific monoclonal antibodies (mAbs). The protective capacity was demonstrated *in vitro* by feeding the mAbs to cultured *F. hepatica*, which impaired fluke survival assessed by reduced motility, fecundity, and reduced active secretion. Furthermore, we could demonstrate a binding of different mAbs to *F. hepatica* gut structures, indicating that liver flukes dine from their environment. Characterisation of the complex gut-associated glycoproteins by a quantitative affinity-enrichment proteomics approach revealed a list of 36 proteins with potential for further vaccine development. Given a conservation of many of these candidates in different *F. hepatica* life stages indicated by available transcriptomic data, such parasite gut-associated glycoproteins serve as a rich source for novel vaccines, directed also against early developmental stages responsible for pathological liver damage.

## 1 Introduction

The parasitic flatworm *Fasciola hepatica*, known as the “common liver fluke,” is a trematode that infects mainly ruminants, causing major economic losses to the agriculture industry worldwide. Importantly, fasciolosis (“liver fluke disease”) has been acknowledged by the World Health Organization (WHO) as a neglected and emerging zoonosis with over 2.4 million infected people in more than 70 countries and several million at risk of infection (Mas-Coma et al., 2018). However, the economical most affected definitive hosts are cattle and sheep, generating more than US$ 3 billion of estimated annual financial losses due to which arise mainly from reduced fertility and loss in milk production, and smaller losses due to anaemia, reduced weight gain, and condemnation of livers at abattoir (Spithill, 1999; Schweizer et al., 2005; Piedrafita et al., 2010; Alvarez Rojas et al., 2015).

Treatment of fasciolosis is based on few anthelmintics with the drug of choice, triclabendazole, because of its ability to kill effectively early immature, juvenile, and adult flukes with an efficacy of over 98% (Boray et al., 1983; Brennan et al., 2007). However, resistant flukes against triclabendazole have been reported in at least 11 countries around the world. Development of anthelmintic resistance is accelerated by incorrect dosing of triclabendazole, and missing pasture and farm management. Furthermore, several cases of humans infected with TCBZ-resistant liver flukes have recently been reported (Kelley et al., 2016). This emphasises the urgent need for new control strategies, including a vaccine as an economically viable alternative control strategy, especially with regard to the subclinical appearance and high prevalence of *F. hepatica* in ruminants irrespective of available drugs.

Over the past few years, many vaccine candidates have been discovered, with the lead *Fasciola* protein candidates cathepsin L, glutathione S-transferase, and fatty acid binding proteins, parts of the parasite’s excreted/secreted products (ESPs) and responsible for intense immune modulation and suppression (Sexton et al., 1990; Dalton et al., 2003; Piedrafita et al., 2004; Toet et al., 2014). Furthermore, the most promising antigen is *Fasciola* spp. leucine aminopeptidase (LAP) tested in sheep. However, highly variable results have been reported with reductions in adult fluke burdens of 49%–89% (Piacenza et al., 1999; Maggioli et al., 2011). LAP is a gut-membrane associated exopeptidase but also detectable in low abundance in the ESP fraction (Acosta et al., 2008).

Gut-associated antigens from tissue dwelling and blood feeding parasites could be a rich source for novel vaccines. These proteins, which are not in contact with the host’s immune system during a natural infection but are vulnerable for antibody-mediated immune attack through a blood and tissue meal, are called hidden antigens (Munn, 1997). Two vaccines have been commercialised against parasite hidden antigens. One of these vaccines, which is no longer available, was TickGARD™ (Willadsen et al., 1989), a vaccine against a membrane-bound glycoprotein of the gut of the blood-feeding tropical cattle tick *Rhiphicephalus (Boophilus) microplus*. Another successful vaccine story against gut-associated glycoproteins is purified native H-gal-glycoprotein and H11 aminopeptidase antigens expressed in the intestine and gut of the blood-feeding nematode *Haemonchus contortus* from sheep, being not entirely “hidden” by a slight antigenic stimulation over few months in a parasite-exposed population (major component of the vaccine Barbervax^®^) (Smith et al., 1994; Knox and Smith, 2001; Smith, 2014). Both examples are native parasite glycoprotein fractions purified by lectins. Lectins are proteins with affinity to carbohydrates and glyco-conjugates, derived from plants, fungi, animals, and microorganisms (Leathem and Brooks, 1998).

Adults and liver migratory larvae of *F. hepatica* are ingesting blood and liver tissue (deliberately or not) during their host residence (Halton, 1967), and—therefore—gut-associated proteins are exposed to antibodies from vaccinated sero-converted individuals. Lectins (carbohydrate-binding proteins) have been shown to bind unspecifically to the gut region of *F. hepatica* (gut lamellae and gastrodermal cells) but also to a range of other “contaminating” parasite somatic tissues (Mcallister et al., 2011). Here, we present a monoclonal-antibody-guided approach to identify gut-associated epitopes based on lectin affinity-purified parasite fractions. We demonstrate an *in vitro* screening of selected antibodies by their interaction with intestinal structures in viable *F. hepatica* flukes, illustrating the relevance of targeted epitopes, and unveil their proteomic identity. Prospectively, the presented proteins are interesting anthelmintic vaccine candidates, either in their purified native conformation or as a glyco-engineered alternative (Naegeli and Aebi, 2015).

## 2 Methods

**Figure 1:**
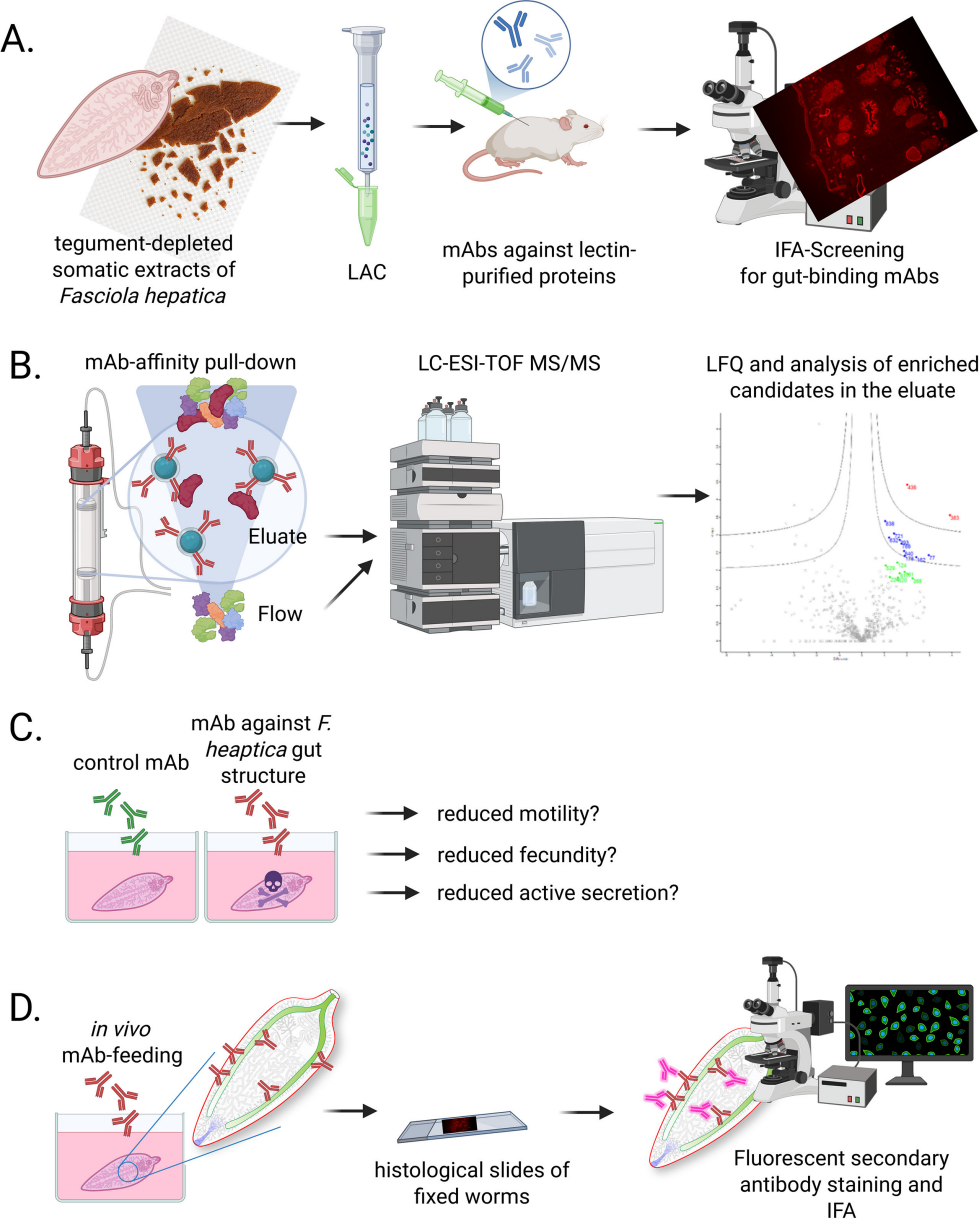
Methodology for the identification and characterisation of *Fasciola hepatica* gut-associated glycoproteins. **(A)** Glycoproteins were purified from tegument-depleted somatic protein extracts by lectin-affinity chromatography (LAC) using five different lectins (ConA, JAC, LEA, PNA, and WGA). Murine monoclonal antibodies (mAbs) were produced against the LAC-glycoprotein fractions and screened for reactivity against *F. hepatica* gut structures by an immune-fluorescence assay (IFA). **(B)** Gut proteins were purified by mAb-affinity pull-down using the previously identified mAbs and further characterised by proteomics. An affinity-enrichment mass-spectrometry approach analysed abundant proteins in the eluate compared with the flow-through by label-free quantitative (LFQ) proteomics. **(C)** *In vitro* flukicide activity of the previously identified mAbs was assessed by a parasite feeding assay with the mAbs followed by the analysis of *F. hepatica* viability by their motility, fecundity, and active protein secretion. **(D)** *In vivo* antibody binding to parasite gut structures was analysed by feeding adult *F. hepatica* with the identified and control mAbs, followed by paralysing and fixing the worms. Gut binding was imaged by direct incubation with fluorescent anti-mouse secondary antibodies and IFA. Figure was created in BioRender (https://BioRender.com).

For the identification and characterisation of *Fasciola hepatica* gut-associated glycoproteins, a combination of lectin-affinity purification of somatic parasite proteins, specifically produced monoclonal antibodies, an affinity-enrichment mass spectrometry pipeline and the assessment of *in vitro* antibody binding and effects on worms was performed. The methodology is summarised in **Figure 1**.

### 2.1 Ethics statement

Experiments with mice were carried out at the experimental units of the Vetsuisse Faculty at the University of Zurich after approval by the Cantonal Veterinary Office of Zurich (permission number ZH235/19) according to Swiss animal rights and regulation standards.

### 2.2 Parasites and parasite protein fractions

Adult liver flukes were collected from naturally infected cattle livers slaughtered at the abattoir in Zurich, Switzerland. Viable parasites were pulled out from the bile ducts and washed five times in sterile phosphate-buffered saline (PBS) and RPMI 1640 (Thermo Fisher) before applying to different approaches (production of excretory/secretory products (ESP) and tegument-depleted somatic extracts).

To produce *F. hepatica* ESP, parasites were placed individually in 12-well plates substituted with 2 mL RPMI 1640 medium containing 2× PSF (Thermo Fisher), 1× L-glutamine (Sigma-Aldrich), 2% D-glucose (Sigma-Aldrich), and 30 mM HEPES (Sigma-Aldrich) and incubated at 37 °C and 5% CO_2_. The media obtained during the first 4 h after parasite culturing were discarded. Viability of the flukes was verified by motility, and viable flukes were placed in fresh medium. ESP were collected after 24 h and centrifuged at 3,000 g for 10 min and stored at −20 °C until used. Only material from viable worms was further used.

Crude somatic extracts were purified from tegument-depleted worms, where tegument stripping was performed according to Wilson et al. (2011) with frozen worms. Subsequently, crude antigen preparation was performed by freeze-thawing and homogenising by sonification (Branson Ultrasonics™ S-250A Sonifier™) in PBS containing 1 mM phenylmethylsulfonyl fluoride (PMSF) (Deplazes et al., 1990). These somatic extracts were further separated into water-soluble proteins (S1), membrane-associated proteins (S2), and detergent soluble proteins (S3) according to Mcallister et al. (2011) by sonication and ultracentrifugation (Beckman Coulter-Optima L-80 XP Ultracentrifuge; SW40Ti rotor).

The protein concentration of all fractions for all experiments was measured using a Pierce BCA Protein Assay Kit (Thermo Fisher). All fractions were kept at −20 °C until further use.

### 2.3 Somatic glycoprotein purification by lectin affinity chromatography

We purified glycoproteins (GPs) by lectin affinity chromatography (LAC) with water-soluble, membrane-associated, and detergent-soluble fractions of tegument-depleted somatic antigens using 5 different lectins (ConA, JAC, LEA, PNA, and WGA) with single column kits using different lectin-agarose beads (Ey Laboratories Inc., and Vector Laboratories), according to the manufacturer’s instructions. The eluted GPs were buffer exchanged and concentrated with PBS using Amicon^®^ Ultra-4 Centrifugal Filters (Merck Millipore Ltd.). For quality control, collected samples were separated by SDS-PAGE (12%) optimised for GPs by boric acid (Sigma-Aldrich) substitution in all buffers and silver stained using SilverQuest™ Silver Staining Kit (Thermo Fisher).

GP fractions were screened for disturbing ESP contamination by a direct ELISA. Briefly, GPs were coated (0.5 μg/well) in coating buffer (0.1 M carbonate/bicarbonate buffer, pH 9.6) and blocked with PBS (pH 7.2) containing 0.02% NaN_3_, 0.05% bovine haemoglobin (Sigma-Aldrich), and 0.3% (v/v) Tween-20 (PBS-T). Detection was based on probing with mouse polyclonal antibodies (pAbs) raised against *F. hepatica* ESP (immunised mouse serum for mAb production; see below). Serum was applied at 1:200 in blocking buffer, 100 μL/well, and incubated for 1 h at 37°C, and bound antibody was detected by alkaline phosphatase-labelled goat anti-mouse IgG (Merck, A3562) at a dilution of 1:10,000 in blocking buffer (100 μL/well, 1 h at 37°C). Absorbance values were read at 405 nm (OD405) after incubation of 100 μL substrate/well of 1 mg/mL PNPP (Sigma-Aldrich) in substrate buffer (0.05 M carbonate/bicarbonate buffer, pH 9.8, containing 1 mM MgCl_2_) at 37°C on a Multiskan RC ELISA reader (Thermo Labsystems).

The fractions purified with ConA and WGA appeared to have a general strong reaction with the anti-*F. hepatica* pAb, which indicated a high ESP contamination (data not shown). Therefore, these fractions were excluded for further approaches. GPs from the water-soluble and membrane-associated somatic antigens in LEA fractions (S1 and S2) showed a weak reaction. This ESP contamination was removed by Protein G Sepharose antibody-affinity chromatography (HiTrap^®^ Protein G HP columns; Merck) according to the manufacturer’s instructions using the anti-*F. hepatica* pAb and negative selection on an ÄKTA pure FPLC (Cytiva) with each 250 µl antibody and 500 µg of LEA-LAC-purified S1 and S2 antigens, respectively.

### 2.4 Production of murine monoclonal antibodies against *F. hepatica* ESP and gut proteins

Monoclonal antibodies (mAbs) were produced similar as described in Eichenberger et al. (2017). A total of 10 NMRI mice (Envigo) were immunised with either *F. hepatica* ESP (four mice), a pooled fraction (1:2) of ESP-depleted S1 and S2, or sole S3 fraction of the LEA-, JAC-, and PNA-LAC-purified eluates (corresponding to “immunisation antigen”). After an initial immunisation with a 100-µl antigen–adjuvant mixture per mouse with 50 µg immunisation antigen mixed 1:2 in Freund’s complete adjuvants (Sigma-Aldrich), two subcutaneous booster injections with immunisation antigens in Freund’s incomplete adjuvants were administered in fortnightly intervals. 10 days after the last injection, a daily intraperitoneal injection of antigen in PBS was administered for 3 days, followed by fusion of murine spleen cells with mouse myeloma cells (Köhler and Milstein, 1975; De Stgroth and Scheidegger, 1980). Only hybridoma fusions, where the polyclonal mouse sera reacted with *F. hepatica* gut structures, as shown by immunofluorescence (IFA) on formalin-fixed paraffin-embedded (FFPE) parasite sections (see below), were selected for further screening.

Specific antibody production of the individual hybridoma cells was screened by ELISA for reactivity against the immunisation antigen and subcloned twice. Furthermore, the positive reacting clones were further verified by IFA (see below) presenting a reaction to *F. hepatica* gut structures. Isotypes were determined using a monoclonal antibody isotyping kit (Sigma Aldrich).

The final selected antibodies based on their gut-binding specificity were purified using ÄKTA™ pure FPLC (Cytiva) with 1 mL HiTrap^®^ IgM Purification HP columns (Cytiva) according to the manufacturer’s instructions. The purified mAbs were dialysed with SnakeSkin^®^ Dialysis Tubing (10,000 MWCO; Thermo Fisher) against PBS containing 0.1 g/L MgCl_2_ and concentrated with Amicon^®^ Ultra-4 Centrifugal Filters (Merck Millipore Ltd.). mAbs were stored at −20°C until further use.

### 2.5 Immune fluorescence assay

Adult flukes were received and washed as described before. Afterwards, they were fixed for 1 min in Berland’s fluid (0.5% formalin in glacial acetic acid) followed by fixation in 10% (v/v) buffered formalin (Berland, 1961; Cooper, 1988). FFPE slides were prepared at the Institute of Veterinary Parasitology, University of Zurich. Deparaffinising tissue rehydration was performed in xylene and a series of ethanol and distilled water, followed by antigen unmasking as described elsewhere (Bartlett et al., 2020). Slides were blocked with 200 µl of 10% goat serum in Tris-buffered saline (TBS) with 0.05% Tween 20 (TBST) for 1 h. The sections were then washed three times in TBST for 5 min and then probed with 200 µl polyclonal antibody or mAb (1:50 in 1% bovine serum albumin (BSA)/TBST) overnight at 4 °C. After rinsing the slides 3 × 5 min in TBST, the sections were incubated with Alexa Fluor (AF) 594 conjugated goat anti-mouse immunoglobulin (H+L) cross-adsorbed secondary antibody (Thermo Fisher) diluted 1:500 in BSA/TBST for 1 h at room temperature in the dark. The sections were washed, and cover slips were applied with antifade mounting medium (Vectashield containing DAPI; Vector Laboratories). The slides were screened on a fluorescence microscope (DMI6000B; Leica). Images were compiled in ImageJ (v1.52p).

### 2.6 Assessment of *in vitro* activity of mAbs against adult *Fasciola* worms

Adult liver flukes were collected as described above. Parasites were placed individually in a 24-well plate in 1.5 mL RPMI 1640 medium containing 2× PSF (Thermo Fisher), 1× L-glutamine (Sigma-Aldrich), 2% D-glucose (Sigma-Aldrich), and 30 mM HEPES (Sigma-Aldrich) substituted with 5% foetal calf serum and incubated at 37 °C and 5% CO_2_. Only parasites which were highly motile after an overnight incubation were further used for the experiments to assess the *in vitro* activity against the mAbs.

After an overnight adaptation to the culture, viable worms were fed with the five different mAbs with binding to gut structures of *F. hepatica*. Initially, a titration experiment was performed to determine an effective concentration of the feeding antibody using three viable adult *F. hepatica* worms. An unrelated mAb of the same isotype (directed against *Cysticercus bovis* crude antigens, unpublished) was included as negative control and 1 µM albendazole as positive control. A concentration of 250 µg mAb per mL medium resulted in consistent findings (**Supplementary Figure 1**). The experiment was repeated with eight viable adult *F. hepatica* worms per condition in a blinded manner.

First, viability by worm motility was monitored 14 h post treatment. Motility of the worms were macroscopically assessed by scoring (3 = normal agile movements, 2 = reduced movement, 1 = very weak movements, which is only visible under the microscope with a 20× objective, and 0 = no movements, i.e., with dead flukes, unable to see any movement for 2 min) (Duthaler et al., 2010; Machicado et al., 2019).

Second, fecundity was assessed by total egg count and percentage of hatching eggs according to Alvarez et al. (2009). Briefly, the medium was centrifuged 600 × g for 10 min, and the supernatant (containing ESP) was collected and stored at −20 °C until further use (see below). The pellet containing the eggs was resuspended with 3 mL tab water, and the total egg number was counted using a light microscope and a Whitlock counting chamber. Thereafter, the eggs were incubated in the dark at 25 °C for 15 to 17 days in a 12-well plate containing 3 mL tab water per well. After the incubation period, the eggs were exposed for 2 h to daylight to provoke the hatching of miracidia. Subsequently, 1 mL of 10% (v/v) buffered formalin was added to stop the hatching process. Eggs were classified using a light microscope in ‘hatched,’ ‘developed,’ and ‘unhatched’ eggs. If possible, at least 150 eggs were counted.

Third, active secretion was analysed by measuring an uncharacterised ESP protein, which correlates with vitality of the worm. For this purpose, a modified sandwich ELISA for the detection of circulating *F. hepatica* antigen was adapted (Langley and Hillyer, 1989). In brief, a purified mAb from mice immunised with ESP (from the same animals as to produce mouse pAb) was coated overnight in a wet chamber (0.5 µg/well). ESP from the *in vitro* viability assays were diluted 1: 20 and 1:100 in PBS containing 0.05% Tween20 (PBST) and added in duplicates (100 µl/well). A biotinylated (EZ-Link™ Sulfo-NHS-Biotin, Thermo Fisher) hyper-immune rabbit serum containing polyclonal anti-*F. hepatica* ESP immunoglobulins (custom produced by a private company; Davids Biotechnologie) was added at a dilution of 1:400 in PBST. Streptavidin-coated alkaline phosphatase was applied (1:2,500 in PBST; Sigma-Aldrich), and absorbance values were read at 405 nm after incubation with 100 μL/well of 1 mg/mL PNPP. Test performance was prior evaluated by measuring different concentrations of fluke ESP, by validation of interactions of screening mAbs in the culture medium, and by correlating fluke motility and ESP production (**Supplementary Figures 2**, **S3**). Final quantification was performed by including a standard curve with known concentration of ESP and comparison of the value from the sample dilution in range.

Statistical evaluation was based on the results from combined data of the duplicate experiments (titration experiment and final experiment, with total n = 11 flukes). For the motility, egg hatching rates, and ESP concentrations, comparisons were made between the sample treatments with the anti-*F. hepatica*-treated and unrelated mAb-treated groups; p values of < 0.05 were considered significant. When two groups were compared, a Mann–Whitney (unpaired, non-parametric) U-test was applied. For the comparison of egg hatching rates in the groups, parametric analysis of variance (ANOVA) plus Tukey’s test were used for the statistical comparison of the egg hatch data obtained from each experiment. The statistical analysis was performed in GraphPad Prism (Version 9.1.0).

### 2.7 Imaging of *in vivo* mAb binding

Viable flukes were cultured as described above and treated with mAbs at a concentration of 250 µg/mL. Two flukes were collected after 1, 2, 4, and 8 h and immersed in Berland’s fluid and fixed in 10% (v/v) buffered formalin. FFPE slides were deparaffinised, unmasked, and blocked as above, before direct incubation with AF 594 anti-mouse antibody and imaging. A feeding control with an unrelated mAb of the same isotype was included (see above).

### 2.8 Affinity-enrichment pull-down proteomics

Antibody affinity chromatography (AC) for the pull-down of mAb binding partners was performed with Protein L-coated beads (Pierce™, Thermo Scientific) according to the manufacturer’s instructions. Optimal concentrations of bead, monoclonal antibody, and antigen were initially evaluated before purifying antigen for the further mass spectrometry analysis. Pull-downs with mAb Fh-mumab-LEA_1 to 4 were performed with pooled LEA-purified S1 and S2 somatic fractions (water-soluble and membrane-associated proteins), and Fh-mumab-PNA_1 with PNA-purified S3 somatic fraction (detergent-soluble proteins).

Eluted protein fractions and various controls to account for unspecific background binders (flow through of the AC, unspecific protein binding to the magnetic beads without coupled mAbs, and two unrelated antibodies of the same isotype (isotype controls with two murine IgM mAbs against *Echinococcus multilocularis*) (Kronenberg et al., 2023)) were further analysed by mass spectrometry. To allow for more efficient enzyme digestion of the protein prior to the LC-MS analysis, the samples were first deglycosylated using the Protein Deglycosylation Mix II (New England Biolabs, Ipswich, USA) according to the manufacturer’s protocol. For mass spectrometry analysis, protein digestion was performed by SP3-assisted digest with carbamidomethylation (Moggridge et al., 2018) with an initial binding on carboxylated magnetic beads in 100% ethanol (v/v) for 30 min at 800 rpm in a ThermoMixer and tryptic digestion (Trypsin/Lys-C mix; Promega) in 10 mM Tris/2 mM CaCl_2_ (pH 8.2) in a 1:50 enzyme to protein ratio. A 30-µg protein per fraction was applied in triplicates. Peptides were cleaned by in-house produced C18 stage tip columns using C18 extraction disks (CDS Empore™; Thermo Fisher). Peptides were diluted in 3% ACN, 0.1% FA to 1 µg/µl, and retention time normalisation peptides (iRT, Biognosys) were added (1:20). Data-dependent analysis (DDA) was performed on an Orbitrap Fusion Lumos Tribrid Mass Spectrometer (Thermo Fisher) operated in line with a nanoACQUITY UHPLC M-class system (Waters).

Per sample, 2 μL was loaded and eluted by running a linear gradient from 5% to 32% solvent B over 90 min (solvent A:0.1% FA in water, solvent B:0.1% FA in ACN) at 300 nl/min. Peptides were ionised utilising a nano-electrospray ionisation (ESI) source (Digital PicoView 565, O/N: DPV-550-565, New Objective, Woburn, MA) and a 10-μm fused-silica spray tip emitter (New Objective, PN). MS1 scans covering 300-2,000 m/z were recorded in profile mode with a resolution of 120,000 using positive polarity and automated gain control (AGC) with a target value of 500,000 and a maximum injection time (maxIT) of 50 ms. Every full MS1 scan was followed by DDA scans recorded in centroid mode with a resolution of 30,000. The AGC target was set to 10,000 and the maxIT to 80 ms. Isolated precursors were fragmented with higher-energy collisional dissociation (HCD) at a normalised collision energy (NCE) of 35%. Fixed first mass was set to 140 m/z.

Identification and label-free quantification (LFQ) of proteins was performed in MaxQuant [version 1.6.17.0; (Cox and Mann, 2008)]. The protein database was built with the *F. hepatica* gene models PRJNA179522 and PRJEB25283 retrieved from WormBase ParaSite [version: WBPS16 (WS279)] appended with common contaminants from an *in house* database substituted with the common repository of adventitious proteins (cRAP; https://www.thegpm.org/crap/). Carbamidomethylation of cysteines was set as fixed modification and methionine oxidation and deamidation of asparagine (N) was included as variable modification. The mass spectrometry proteomics data have been deposited in the ProteomeXchange Consortium via the PRIDE partner repository with the dataset identifier PXD035194 and 10.6019/PXD035194.

Further analysis of enriched proteins in the corresponding eluates was based on the protein group intensity values from MaxQuant, where LFQ intensity values were used to compare the abundance of identified proteins between the different fractions. A final evaluation of the mAb–protein interaction was performed in Perseus (version 1.6.15.0; (Tyanova et al., 2016) according to Keilhauer et al. (2015), with some modifications. In brief, only proteins identified with a minimum of two peptides and in three replicates were further included, analysed per group separately. LFQ intensities were log_2_ transformed. A Student’s t-test was performed comparing the bait (eluate) and the flow-through, where hits with the background (group of unspecific binding and two isotype controls) were previously depleted from the dataset. The p values were corrected for multiple testing by Benjamini and Hochberg (Benjamini and Hochberg, 1995). If not stated otherwise in the results, volcano plots were generated in Perseus with S0 = 0.1 and FDR = 0.01 as class 1 parameters (more stringent) and FDR = 0.05 as class 2 parameters (less stringent). The mean of the three biological replicates was taken as the calculated abundance represented as mean intensities.

### 2.9 Immunoreactivity of affinity-enriched protein fractions against sera of naturally infected ruminants

Antibody-enriched protein fractions applied in the pull-down proteomics approach were further analysed by ELISA for their absent reactivity with sera from naturally infected dairy cows. Sera from cattle were collected at abattoir, and bile was taken from gallbladders and examined for the presence of *F. hepatica* eggs (Eichenberger et al., 2013). Antibody status of 10 positive and 10 negative sera was further confirmed by a commercial Fasciolosis Verification Test (IDEXX).

Microtitre plates (Nunc MaxiSorb) were coated overnight with the five different affinity-purified GP fractions (0.5 µg/well), blocked with PBS (pH 7.2) containing 0.02% NaN_3_, 0.05% bovine haemoglobin (Sigma-Aldrich), and 0.3% (v/v) Tween-20 (PBS-T). After three washes, the plates were incubated with sera (1:100 in blocking buffer) for 1 h at 37°C. Unbound antibodies were removed by washing three times, and the plates were incubated with alkaline phosphatase-conjugated secondary antibodies (goat anti-bovine IgG 1:2000; KPL #5220-0383) for 1 h at 37°C. The plates were washed four times, and absorbance values were read at 405 nm after incubation with substrate (PNPP).

As a coating control, the same GP fractions were also probed with the previously purified murine mAbs against the lectin-purified *F. hepatica* antigens and alkaline phosphatase-labelled goat anti-mouse IgG (Merck, A3562) at a dilution of 1:10,000 in blocking buffer.

### 2.10 Functional characterisation, *in silico* analysis of glycosylation, and transcriptomic analysis of identified protein hits

NetNGlyc 1.0d execution was downloaded from the NetNGlyc server (Gupta and Brunak, 2002) and used to determine potential N-glycosylation sites, trained with *F. hepatica* tegumental glycoproteins with potential glycosylation sides [extracted from Ravidà et al. (2016)]. Transmembrane helices in proteins were predicted by the TMHMM 2.0 server (Krogh et al., 2001). The SignalP 6.0 server was used to predict signal peptide for secretory proteins (Nielsen et al., 2019). Functional annotation of proteins was achieved by gene ontology mapping through Blast2GO 6.0.3 (Götz et al., 2008). Subcellular localisation was performed in the WoLFPSORT prediction (Horton et al., 2007). Top hits identified by affinity-enrichment mass spectrometry were mapped to the published transcriptomic and somatic proteomic datasets for the liver migratory larval life stage (Cwiklinski et al., 2021). Means from the triplicate values were recorded. Furthermore, the protein hits were blasted (NCBI Blast + executables v2.15.0) against the list of characterised glycoproteins from newly excysted juveniles (De Marco Verissimo et al., 2021).

## 3 Results

### 3.1 Monoclonal antibody-guided screening for *Fasciola hepatica* antigens reveal specific gut proteins

**Figure 2:**
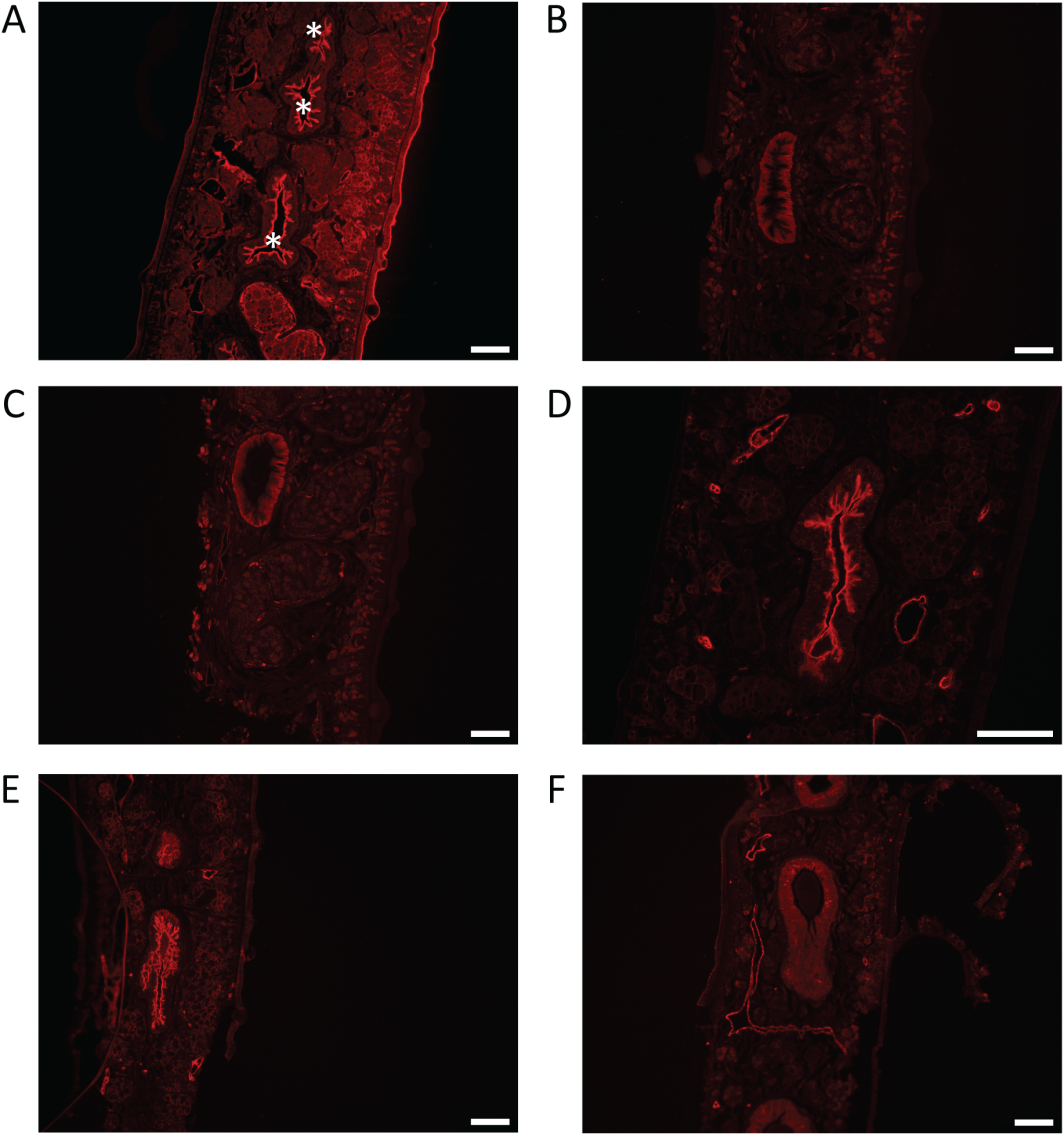
Monoclonal antibodies against lectin-affinity-purified *Fasciola hepatica* somatic extracts immuno-localise with gut structures in transversal worm sections. **(A)** Reaction of polyclonal serum of a mouse immunised against *F. hepatica* excretory/secretory products. Asterisks (*) indicate the gut of the fluke, where the lamellae are shining bright, surrounded by a light signal of the gastrodermis. **(B, C)** Reaction of monoclonal antibody (mAb) Fh-mumab-LEA_1 and 2 to gastrodermal cells, respectively. **(D, E)** Reaction of mAb Fh-mumab-LEA_3 and 4 to gut lamellae, respectively. **(F)** mAb Fh-mumab-PNA_1 reacts to gastrodermal cells but also shows background signal with other tissues. A secondary antibody control (AF594 goat anti-mouse immunoglobulin) was performed without any signal (not shown). Bar corresponds to 100 µm.

We purified glycoproteins by lectin affinity chromatography (LAC) with water-soluble, membrane-associated, and detergent-soluble fractions of tegument-depleted somatic antigens using five different lectins (ConA, JAC, LEA, PNA, and WGA). The fractions purified with ConA and WGA showed strong contamination with ESP and were excluded for further approaches. First, mAbs were produced against different LAC-purified fractions and screened by ELISA for specific recognition of the immunisation antigen, followed by a confirmation assay by specific gut binding in an immune fluorescence assay (IFA). In the screening process, many monoclonal antibodies reacted unspecifically and against reproductive structures, and five mAbs with specific binding to the gastrodermis (Fh-mumab-LEA_1 and Fh-mumab-LEA_2) or the lamellae of the *F. hepatica* gut (Fh-mumab-LEA_3, Fh-mumab-LEA_4, and Fh-mumab-PNA_1) were identified (**Figure 2**).

### 3.2 Antibodies against *Fasciola hepatica* gut-associated antigens reduce parasite fitness in an *in vitro* parasite culture system

The protective efficacy of the antibodies was evaluated by incubation and treatment of living adult flukes with the purified mAbs compared with a control group with unrelated mouse mAb of the same isotype (all IgM). Viability was assessed by motility scoring, fecundity assessment (*F. hepatica* total egg count and egg development), and evaluation of active secretion by measuring secreted ESP concentrations.

**Figure 3:**
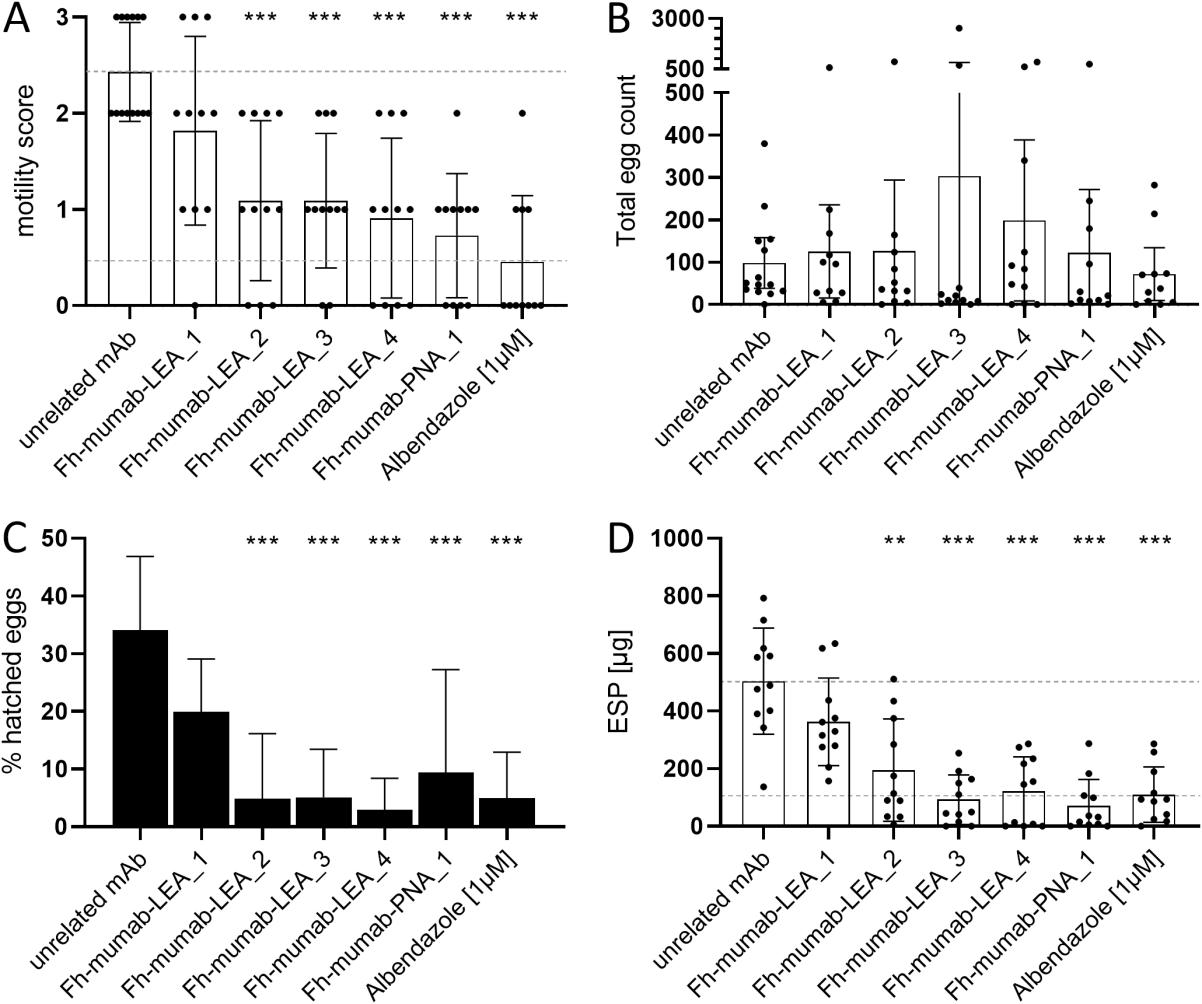
Monoclonal antibodies against *F. hepatica* hidden and gut-associated proteins reduce worm fitness, fecundity, and metabolic product in an *in vitro* viability assay. **(A)** Motility score for viability. **(B)** Total egg production. **(C)** Relative amount of hatched miracidia from the eggs after 15 days as an indicator for parasite fecundity. **(D)** Excretory/secretory products (ESP) in the culture supernatant after 14 h post treatment. Data of 11 flukes per group are compared with the feeding of an unrelated negative control (**p < 0.01; ***p < 0.001). Albendazole (1 µM) was included as a treatment control.

First, a titration experiment was performed to determine an effective concentration of the feeding antibody. A concentration of 250 µg mAb per mL medium resulted in consistent findings (**Supplementary Figure 1**). Repeated experiment was performed in a blinded format, where four of the five mAbs (Fh-mumab-LEA_2 to 4 and Fh-mumab-PNA_1) showed significant reduction in motility, egg hatching, and measurement of the ESP (**Figure 3**). The total egg count, however, was not significantly different from the negative control group and the albendazole treated group in any of the treated flukes.

**Figure 4:**
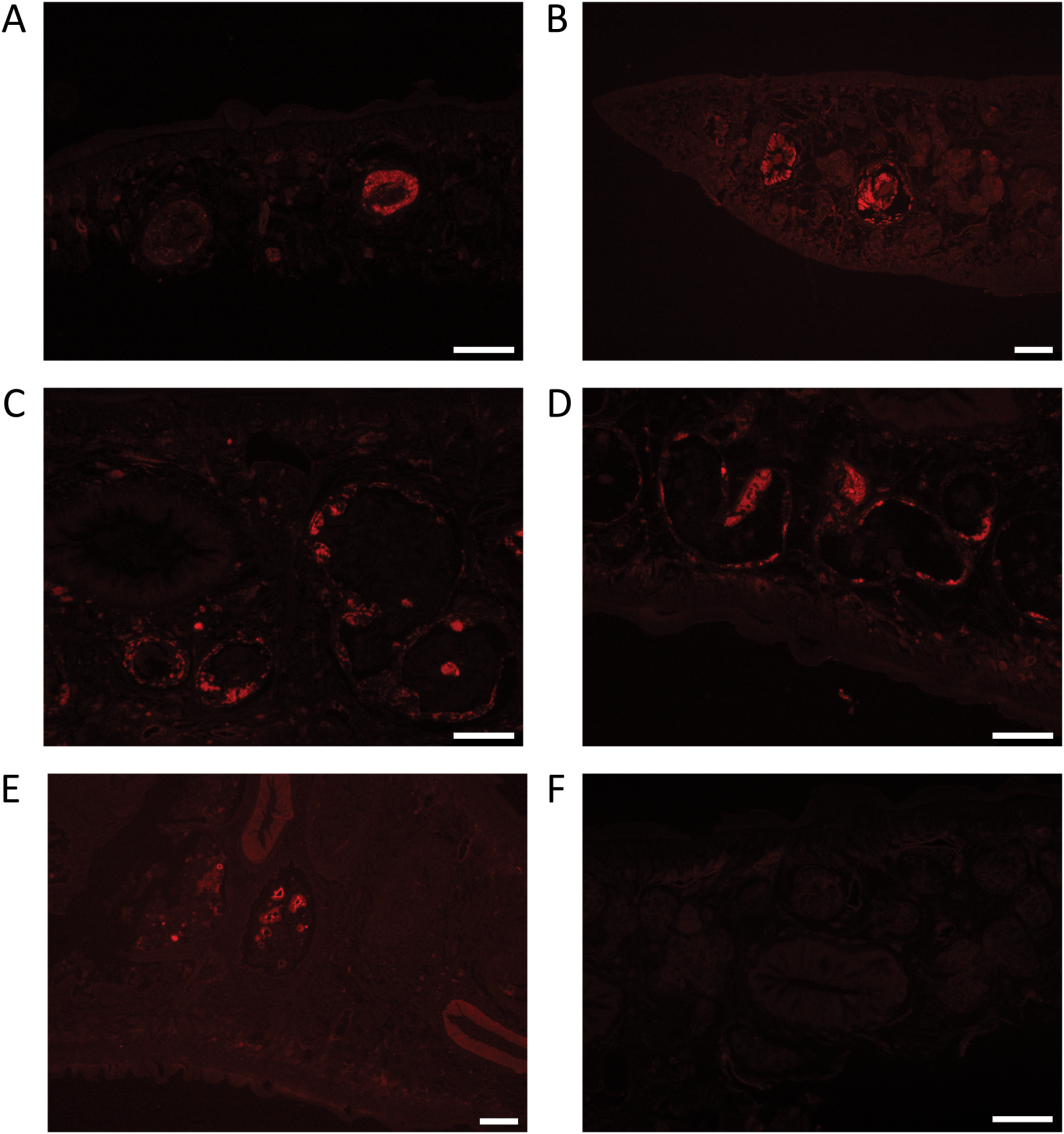
Binding of antibodies to intestinal structures of viable *F. hepatica* worms after *in vivo* feeding and further probing with anti-mouse IgG conjugated to AF-594 in transversal sections. mAb Fh-mumab-LEA_1 and 2 are binding to the worm gastrodermal cells **(A, B)**, whereas Fh-mumam-LEA_3 and 4 **(C, D)** are binding to the gut lamellae. Fh-mumab-PNA_1 **(E)** shows a mixed reaction. A feeding control with an unrelated monoclonal antibody of the same isotype was included **(F)**. Bar corresponds to 100 µm.

We demonstrated a binding for all the mAbs to the lamellae or gastrodermis of the gut by *in vitro* feeding of the flukes with the mAbs, followed by FFPE-slide preparation and IFA. While after 1 and 2 h of incubation no binding of the mAbs could be detected, a similar affinity to the tissue as in the screening approach was imaged starting after 4 h post treatment (**Figure 4**). Therefore, we concluded that the selected mAbs are ingested by *F. hepatica* from the surrounding culture media.

### 3.3 Quantitative pull-down proteomics identified a list of parasite-stage conserved gut-related proteins

To uncover and purify the proteins from liver fluke extracts, we performed immunoprecipitation by magnetic beads-based antibody affinity chromatography and a modified ‘affinity-enrichment mass spectrometry’ pipeline. First, antibody-affinity-purified protein fractions with the five different mAbs were analysed for an absence of immunoreactivity with naturally infected cattle. All included sera (from *F. hepatica*-infected and uninfected animals) showed negative results with optical densities (OD values) between 0.01 and 0.06, whereas a binding control with the corresponding mAbs showed values of 1.13 to 1.67.

**Figure 5:**
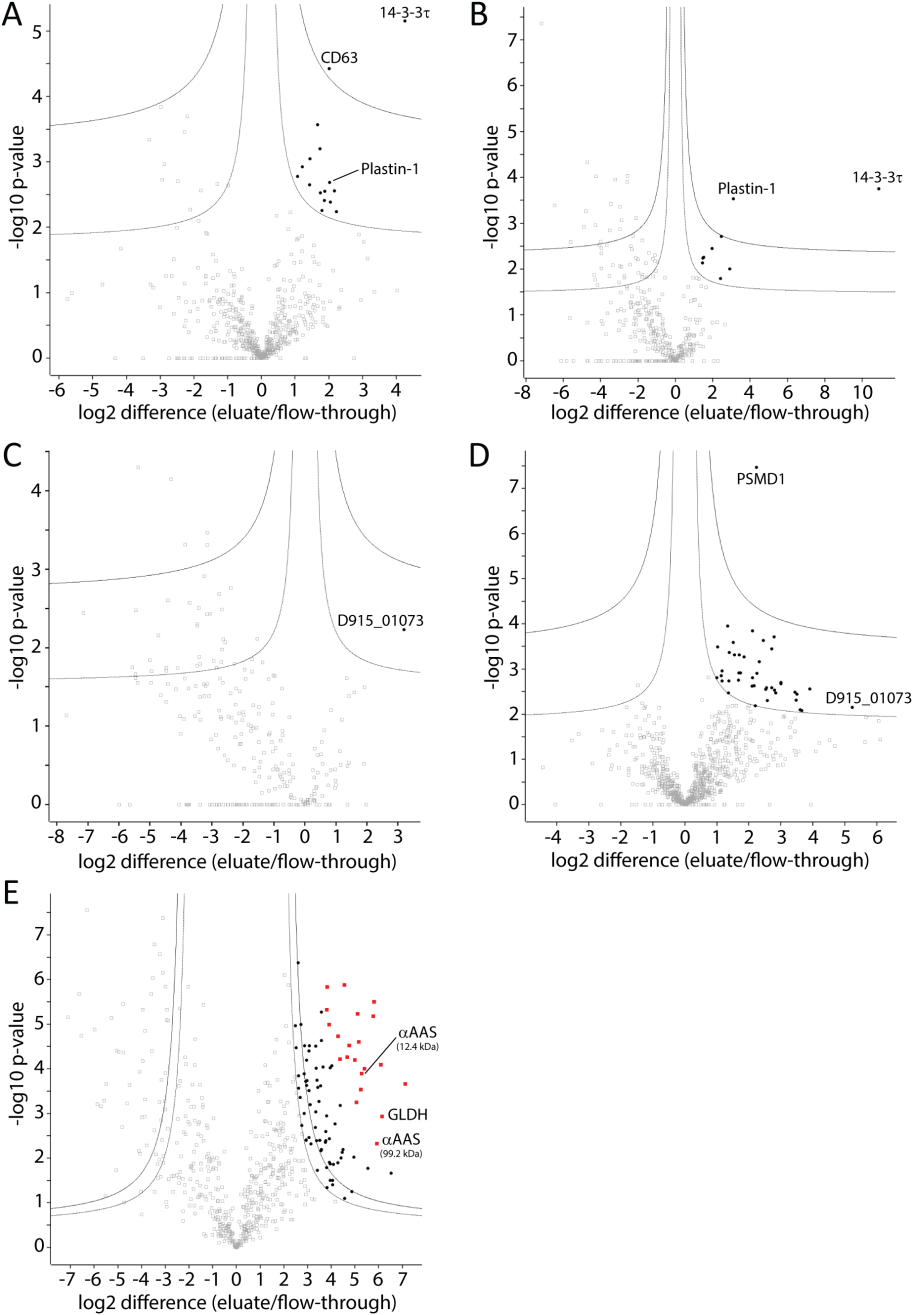
Label-free quantitative mass spectrometry approach with affinity-enriched fractions shows relative more abundant proteins bound to the antibodies as compared with the unbound fractions. Bait was magnetic beads coupled with mAb-Fh-mumab-LEA1 to 4 **(A-D)** or mAb-Fh-mumab-PNA1. The inner line represents p < 0.05, the outer line represents p < 0.01, and filled squares **(E)** correspond to more stringent parameters with p < 0.001. Discussed candidates are highlighted.

By affinity-enrichment pull-down proteomics, most abundant protein hits for Fh-mumab-LEA_1 and 2 each comprise the N-glycosylated “tyrosine 3-monooxygenase/tryptophan 5-monooxygenase activation protein theta polypeptide” belonging to the 14-3–3 tau protein family (**Figure 5**). The predicted protein size of 36.2 kDa is consistent with an observed smeared protein band in pull-down optimisation experiments and quality control by SDS-PAGE (**Supplementary Figure 4**). Furthermore, “23 kDa integral membrane protein” could be identified for Fh-mumab-LEA_1 and the actin-binding protein “plastin-1” for Fh-mumab-LEA_2, respectively, although less abundant. Fh-mumab-LEA_3 and 4 both detected a so far uncharacterised “hypothetical protein” (D915_01073). This was the only significant hit for Fh-mumab-LEA_3. Although less significant, this hypothetical protein was also the most abundant protein in the eluate of the Fh-mumab-LEA_4 pull-downs.

For the Fh-mumab-PNA_1 pull-down, 31 hits were identified, although more stringent parameters were applied to analyse the proteomic data (for example by increasing the S_0_ value, which is in essence a minimal fold change between the compared groups, which provides significant p-values based on a selected FDR of below 1‰). Top 10 hits were further analysed by additional information on potential subcellular location and label-free quantitative intensity in the mass spectra (summarised in **Supplementary Table 1**). The SDS-PAGE picture performed as quality control after magnetic bead pull-down showed a vague molecular size range of a smear between 20 and 45 kDa and a visible band in the high molecular range of >100 kDa. Top *F. hepatica* protein hits in this range were glutamate dehydrogenase, tubulin, propionyl-CoA carboxylase alpha chain, and clathrin, an uncharacterised “hypothetical protein” (D915_003331). Interestingly, an enzyme involved in the breakdown of lysine (alpha-aminoadipic semialdehyde synthase) shows up twice—once in a canonical high-molecular form (99 kDa) and once in a small (12 kDa) membrane integrated form.

The expression levels by transcript abundances (FPKM) in juvenile worms of the top proteomic hits indicate a conserved expression profile of the identified gut proteins. Furthermore, most of the proteins could be identified in the somatic proteome of 21-day-old immature *F. hepatica* flukes. A summary of all significant hits, along with BLAST results against the published glycoproteomic dataset, is provided in **Table 1** and **Supplementary Table 1**.

**Table 1:** List of potential *Fasciola hepatica* hidden and gut-related proteins identified by quantitative pull-down proteomics.

Functional annotation	Mol. weight [kDa]	LFQ signal intensity	N-glycosylation sites	Transmembrane helixes	Signal peptide	FPKM in liver juveniles
Fh-mumab-LEA_1						
14-3-3τ	36.2	1.70E+08	2	0	0	1193.9*
CD63	20.5	7.72E+06	0	4	0	952.5*
Fh-mumab-LEA_2						
14-3-3τ	36.2	1.53E+08	2	0	0	1193.9*
Plastin-1	56.4	5.61E+06	1	0	0	0
Fh-mumab-LEA_3						
D915_01073	12.6	9.13E+08	3	0	0	63.9
Fh-mumab-LEA_4						
PSMD1	141.9	6.07E+07	1	0	0	60.2*
Fh-mumab-PNA_1						
PCCB	58.6	2.06E+10	1	0	0	739.8*
GLDH	23.2	1.25E+10	2	0	0	2319.2*
αASS	99.2	1.04E+10	5	0	0	302.2*
Tubulin α1C	99.3	7.04E+08	0	0	0	1048.9*
PCC	35.2	6.84E+08	1	0	0	800.3*
RPS13	17.1	4.98E+08	0	0	0	758.6*
αASS	12.4	4.93E+08	1	1	0	302.2*
CLTC	194.3	4.92E+08	6	0	0	87*
D915_003331	95.2	3.41E+08	5	0	0	7.6*
ATP-PFK	80.1	3.23E+08	1	0	0	102

Immunoprecipitation by magnetic bead-based antibody affinity chromatography and a modified ‘affinity-enrichment mass spectrometry’ pipeline revealed significant hits for the different monoclonal antibodies (mAbs). For mAb Fh-mumab-PNA_1, only the top 10 significant hits are shown. The complete list is summarised in **Supplementary Table 1**. Proteins which are present in the somatic proteome of 21-day-old immature flukes are marked by an asterisk (*). LFQ, label-free quantification measured in MaxQuant; FPKM, fragments per kilobase per million mapped reads, as a measure of mRNA expression in liver-migrating juveniles [stage-specific transcriptomic data from Cwiklinski et al. (2021)].

## 4 Discussion

*Fasciola hepatica* is the zoonotic infectious agent responsible for liver fluke disease in livestock, causing substantial economic burden. Coevolution of the fluke with its host resulted in a balanced relationship with a pronounced humoral response (mainly against glyco-moieties), which is not protective (Chauvin et al., 1995; Clery et al., 1996; Mulcahy et al., 1998). Furthermore, a global emergence of anthelmintic resistant flukes emphasises the urgency of novel control strategies, where vaccines could be a viable strategy (Beesley et al., 2018).

This work presents a lectin- and mAb-guided purification and subsequent characterisation of gut-associated proteins, which could be a rich source of novel vaccine candidates to control fasciolosis. The fluke’s ability to evade and modulate the host’s immune system by ESPs and surface molecules, responsible to establish chronic infections, conversely hampers vaccine success by conventional protein candidates (Loukas and Giacomin, 2015). In this sense, potentially hidden antigens as immunogenic agents, which theoretically are not subjected to an evolutionary pressure for the parasite to generate an immune evasion, should circumvent host evasion by the parasite and induce a protective antibody response against the tissue dwelling and blood feeding liver fluke.

Hidden and somatic gut antigens from *F. hepatica* cannot easily be purified by lectin affinity chromatography (LAC), as compared for example to the commercially available ‘holistic’ vaccine against the gastrointestinal nematode *Haemonchus contortus* in sheep based on intestinal antigens by ConA-purified worm extracts (Smith et al., 1993; Mcallister et al., 2011). Inspired by a previous demonstration of lectins binding unspecific to intestinal and other tissues of liver flukes (Mcallister et al., 2011), we purified specific gut-associated glycoproteins by LAC and antibody-AC with newly produced mAbs. These mAbs against gut proteins were further used to characterise their protective capacity. For this purpose, we mimicked a specific humoral response against fluke gut antigens by feeding antibodies, which impaired the fitness of the parasite by reduced motility, fecundity, and active secretion *in vitro*. Indeed, we demonstrated that flukes dine the antibodies from the culture medium visualised by imaging approach. Hence, there is strong indication that the diet of anti-gut antibodies by flukes leads to the identification of feasible anti-helminth vaccine candidates. However, the putative ‘hidden’ nature of these protein candidates remains to be demonstrated, although their lack of reactivity with sera from naturally infected cows provides supportive evidence.

Five specific mAbs directed against gut-associated antigens of *Fasciola hepatica* (lamellae and gastrodermal cells) were characterised by immunoprecipitation and quantitative proteomics, leading to the identification of 36 target proteins. While some of the mAbs precipitated different proteins (**Figure 5**), the co-elution of additional proteins may reflect their involvement in an essential gut protein network, consistent with the protective effects observed when antibodies were fed to the flukes.

The list of newly identified gut-associated liver fluke proteins also overlaps with previously discussed vaccine candidates. Thereby, promising candidate antigens in the water-soluble and membrane-associated fractions (purified by mAbs against the LEA-AC) are a 14-3–3 zeta family protein (tyrosine 3-monooxygenase/tryptophan 5-monooxygenase activation protein theta polypeptide), and a CD63-protein (tetraspanin). In contrast, *F. hepatica* plastin-1 and an uncharacterised protein containing a conserved domain of unknown function (DUF544), although detected here, are not present in the somatic proteome of early juvenile stages (Cwiklinski et al., 2021).

Notably, 14-3–3 proteins and tetraspanins are abundant in extracellular vesicles (EVs), which are secreted by a wide range of cells and play important roles in intercellular signalling and parasite–host interactions (Andrade et al., 2004; Eichenberger et al., 2017). Helminth EVs and the structural EV-protein ‘tetraspanin’ are discussed to be promising vaccine candidates against various helminths, with 30%-60% reduction in parasite burdens tested in rodent model organisms (Dakshinamoorthy et al., 2013; Mekonnen et al., 2018; Drurey et al., 2020). 14-3–3 isomeres have been shown to be promising vaccine candidates for *Schistosoma* spp. and *Echinococcus* spp (Siles-Lucas et al., 2008), with over 97% reduction in metacestode load in an *E. multilocularis* challenge experiment using recombinant versions of the protein (Siles-Lucas et al., 2003), and various protection against *Schistosoma* spp. of 25%–65% reduction in adult worm burden depending on the expression system (Schechtman et al., 2001; Siles-Lucas et al., 2007). The 14-3–3 protein family display many isoforms, which are expressed in different tissues (Schechtman et al., 2001; Chaithirayanon et al., 2006). For example, a 14-3–3 protein was also detected on the tegument of *F. hepatica* interacting with ruminant-specific glycan-binding proteins (host-derived lectins), critical in host–parasite interactions (Swan et al., 2019). Therefore, different versions of this protein may display different promise for vaccine design. However, the conserved nature of such proteins with various isoforms and potential homologue epitopes with host proteins has to be carefully evaluated in the process of designing a vaccine.

In the different protein fractions, a substantial number of contaminating proteins—including ribosomal, mitochondrial, and nuclear proteins—were co-purified. The mild conditions applied in the pull-down approach may partly account for the broad range of eluted candidates in the detergent-soluble fraction (purified by the mAb against the PNA-AC), particularly due to interactions with sticky glycoproteins and lipophilic membrane proteins. Nevertheless, top 10 abundant proteins revealed plausible gut proteins, including the knowledge on functional data, glycosylation, and transmembrane domains. The list contained glutamate dehydrogenase (GLDH), a gut protein, which for example was discussed as a vaccine candidate against the gastrointestinal nematode *H. contortus* in sheep, but unfortunately with limited success in vaccination experiments (Skuce et al., 1999). Another highly abundant protein and the likely targeted protein based on the molecular weight prediction in the mAb pull-down against the detergent-soluble fraction was alpha-aminoadipic semialdehyde synthase (αASS). αASS is an intestinal protein found in various animal species involved in lysine degradation. Interestingly in the top 10 abundant candidates, proteomics revealed also a 12-kDa homologue with an identical C-terminal part but containing a transmembrane domain. Hence, functional characterisation and potential moonlighting functions essential for parasite survival have to be further investigated.

All but one mAb reduced the viability of adult *F. hepatica* in an *in vitro* feeding system with parasites collected from bile ducts of infected livers at abattoir. As a control, we included albendazole, a drug recommended for the treatment of mature *F. hepatica* worms (over 14 weeks post infection). Notably, not all worms included were affected by the drug treatment, indicating either a mixed population or resistance. Overall, the monoclonal antibody treatment was not as effective as the drug control. Furthermore, data are missing on the effect of these antibodies against juvenile parasites, which still has to be validated.

Attack of liver flukes by a vaccine would be most economical before any liver damage emerges. Because the antibodies showed a negative impact on the viability of the parasites in the *in vitro* feeding of adults, we were interested if the detected protein candidates are also expressed in other parasite life stages. In this sense, a protein which is conserved and expressed among different life stages signifies a promising vaccine candidate. Comparison of the pull-down hits to the published liver-migrating juvenile stage-specific transcriptomic data (Cwiklinski et al., 2021) showed that the proteomic candidates are also expressed in early life stages of *F. hepatica*. Only a few of these proteins—notably tetraspanin—however can be found in a glyco-proteomics approach from crude somatic extracts of newly excysted juveniles (De Marco Verissimo et al., 2023).

In conclusion, our aim was to identify hidden and gut-associated antigens from *F. hepatica* as promising vaccine candidates with an alternative screening system. There, we demonstrated that the combination of protein fractionation, antibody-guided screening, and quantitative mass spectrometry, combined with an initial *in vitro* screening, is a useful tool to identify novel protein candidates. The interaction of the selected antibodies against the detected proteins and the effective protective mechanism is still obscure. Whether this is by tackling an essential protein for nutrition uptake and processing, interaction with cell signalling, and a general blockage of gut functions is currently only speculative and needs further investigation. However, the here described candidate antigens—solely or in combination—should be considered in future vaccine studies in natural hosts or rodent models.

## Data Availability

The datasets presented in this study can be found in online repositories. The names of the repository/repositories and accession number(s) can be found in the article/**Supplementary Material**.

## Supplementary material

Supplementary Figure 1 Dose-response curve of monoclonal antibodies (mAbs) against *Fasciola hepatica* gut-related antigens. Adult worms were exposed in triplicates to three different concentrations of mAbs (50, 125, and 250 µg/mL culture media) and motility was assessed after 14 hours post treatment (Duthaler et al., 2010; Machicado et al., 2019). An unrelated mAb (250 µg/mL) and Albendazole (1 µM) were included as a negative and positive treatment controls. Curve was fitted by non-linear regression. Error bars correspond to SEM.

Supplementary Figure 2 Measurement of a *Fasciola hepatica* ESP protein by a monoclonal antibody (mAb)-based approach. Different concentrations of fluke ESP were applied to the test in duplicates. Validation of potential interactions with the screening antibodies were excluded by adding 25 µg mAb to each condition, where the measurements for the ESP quantification highly correlates (Pearson R^2^ to ESP; Fh-mumab-LEA_1: 0.983; Fh-mumab-LEA_2: 0.981; Fh-mumab-LEA_3: 0.992; Fh-mumab-LEA_4: 0.992; Fh-mumab-PNA_1: 0.995).

Supplementary Figure 3 *Fasciola hepatica* ESP protein measured by a monoclonal antibody (mAb)-based approach correlates with worm survival. ESP of three individual flukes were measured over time in culture. Culture medium was daily replaced, centrifuged (3,000 x g for 10 min), and supernatant stored at -20°C until further analysed. Dead flukes assessed by motility (score = 0) is indicated by a cross (†).

Supplementary Figure 4 Band pattern of pulled-down eluates with different monoclonal antibodies. SDS-PAGEs optimised for glycoproteins followed by silver staining (SilverQuest™, Invitrogen) were performed with eluates from antibody pull-down approaches. Arrows indicate the expected/predicted molecular size based on the amino acid sequence (without posttranslational modification like glycosylation) of most significant and abundant proteins identified (**Table 1** and **Supplementary Table 1**). Lane 1: Fh-mumab-LEA_1; Lane 2: Fh-mumab-LEA_2; Lane 3: Fh-mumab-LEA_3; Lane 4: Fh-mumab-LEA_4; Lane 5: Fh-mumab-PNA_1. Common bands from co-eluted IgM-antibodies are Ig-light and heavy chains (approx. 27 and 72 kDa), J-chain (approx. 15 kDa), a precipitate of J- and light chain (approx. 42–45 kDa), and partially reduced IgM-immunoglobulins (smear around 100 kDa). A pre-stained protein ladder (PageRuler 26616, Thermo Fisher) was used as molecular marker.

Supplementary Table 1 Significant protein hits from an affinity-enrichment pull-down proteomics approach using the 5 monoclonal antibodies.
