# Supplementary figures and images for: Identification of Fasciola hepatica gut-associated glycoproteins as potential vaccine candidates by lectin-affinity chromatography, flukicidal monoclonal antibodies, and affinity-enrichment mass spectrometry
Source: Front Parasitol. 2026 May 29;5:1746419. doi: 10.3389/fpara.2026.1746419 (PMC13260064; doi:10.3389/fpara.2026.1746419)

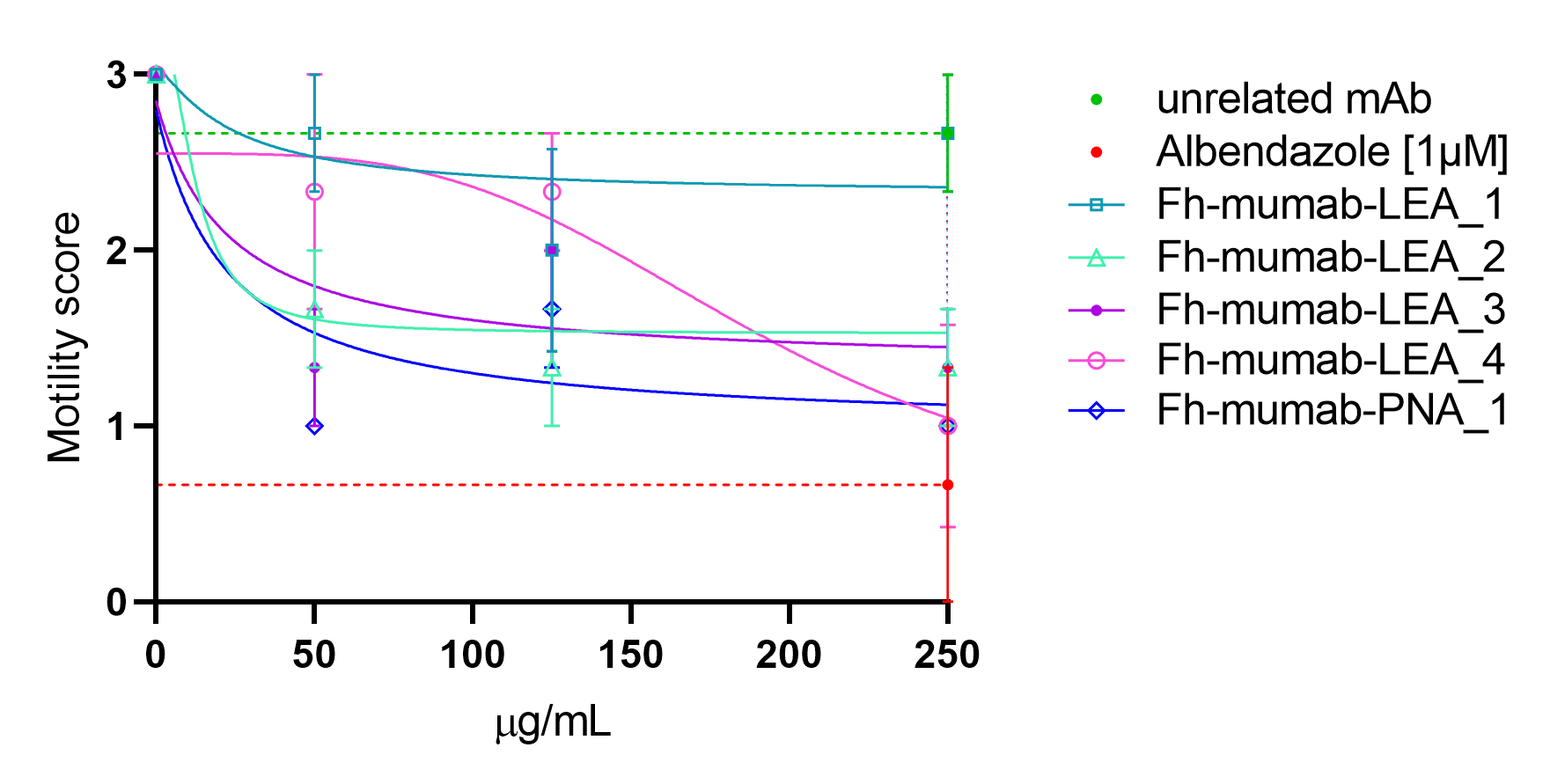

Supplement: Supplementary Figure 1 — Dose-response curve of monoclonal antibodies (mAbs) against Fasciola hepatica gut-related antigens. Adult worms were exposed in triplicates to three different concentrations of mAbs (50, 125, and 250 µg/mL culture media) and motility was assessed after 14 hours post treatment (Duthaler et al., 2010; Machicado et al., 2019). An unrelated mAb (250 µg/mL) and Albendazole (1 µM) were included as a negative and positive treatment controls. Curve was fitted by non-linear regression. Error bars correspond to SEM. [file Image1.tif]

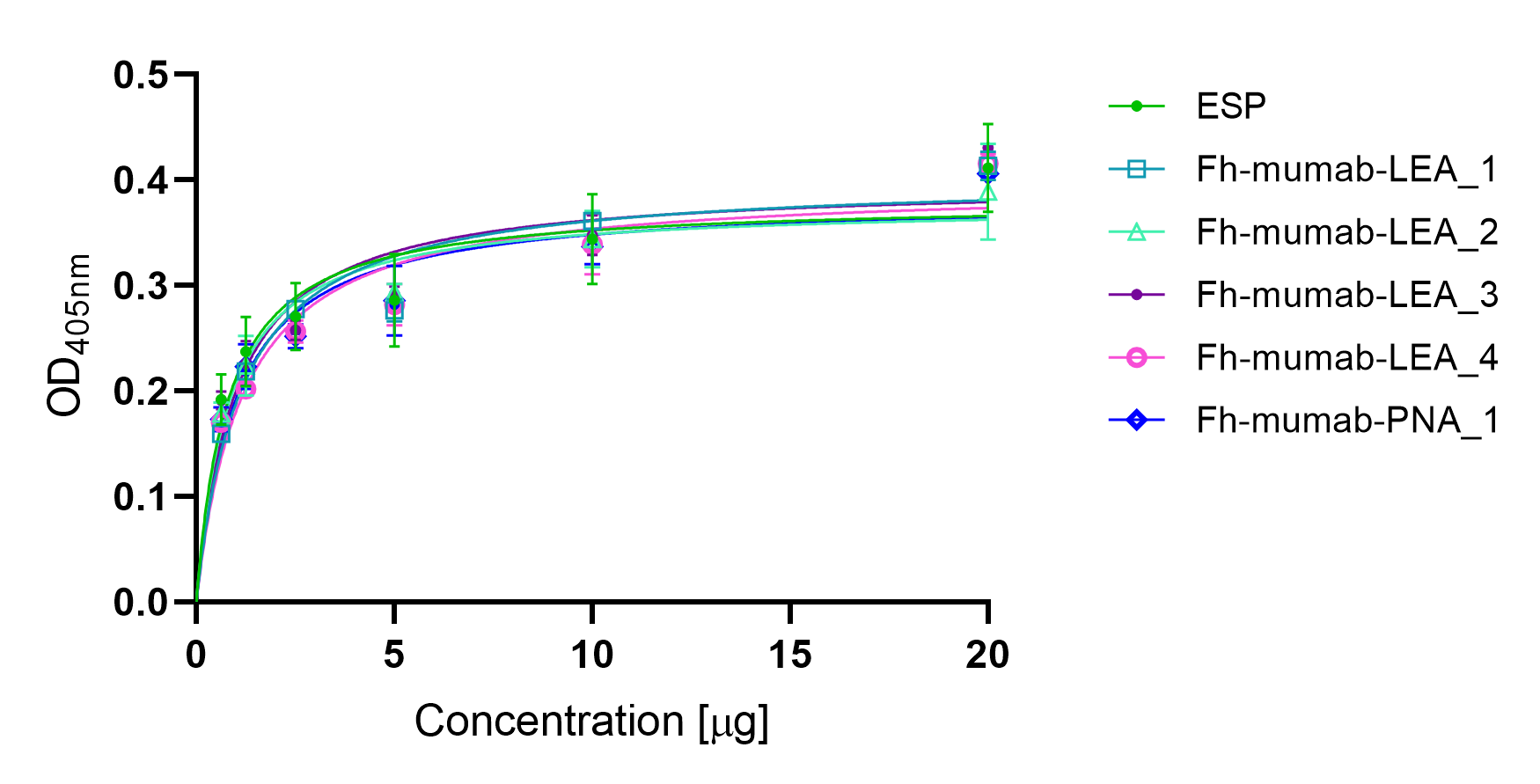

Supplement: Supplementary Figure 2 — Measurement of a Fasciola hepatica ESP protein by a monoclonal antibody (mAb)-based approach. Different concentrations of fluke ESP were applied to the test in duplicates. Validation of potential interactions with the screening antibodies were excluded by adding 25 µg mAb to each condition, where the measurements for the ESP quantification highly correlates (Pearson R2 to ESP; Fh-mumab-LEA_1: 0.983; Fh-mumab-LEA_2: 0.981; Fh-mumab-LEA_3: 0.992; Fh-mumab-LEA_4: 0.992; Fh-mumab-PNA_1: 0.995). [file Image2.tif]

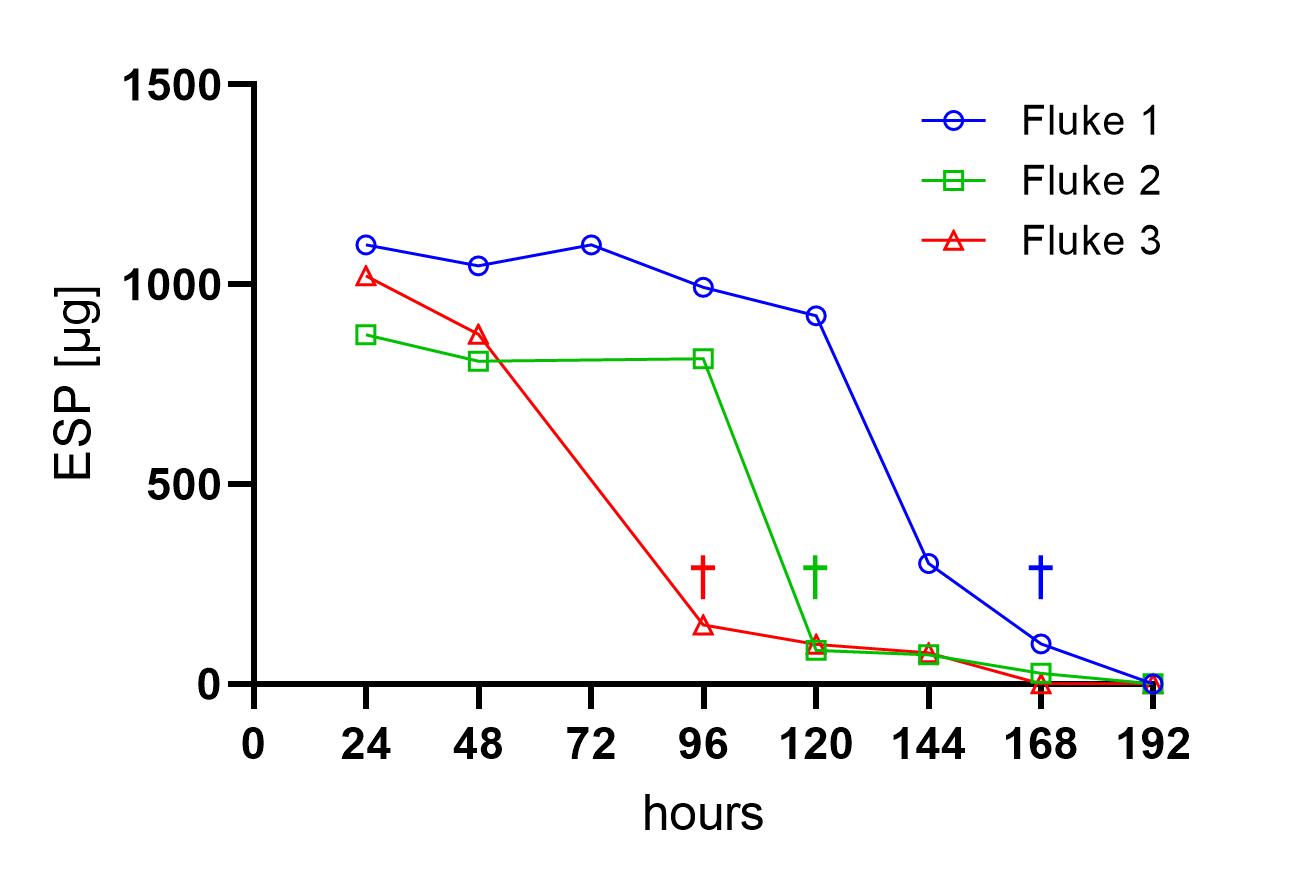

Supplement: Supplementary Figure 3 — Fasciola hepatica ESP protein measured by a monoclonal antibody (mAb)-based approach correlates with worm survival. ESP of three individual flukes were measured over time in culture. Culture medium was daily replaced, centrifuged (3,000 x g for 10 min), and supernatant stored at -20°C until further analysed. Dead flukes assessed by motility (score = 0) is indicated by a cross (†). [file Image3.tif]

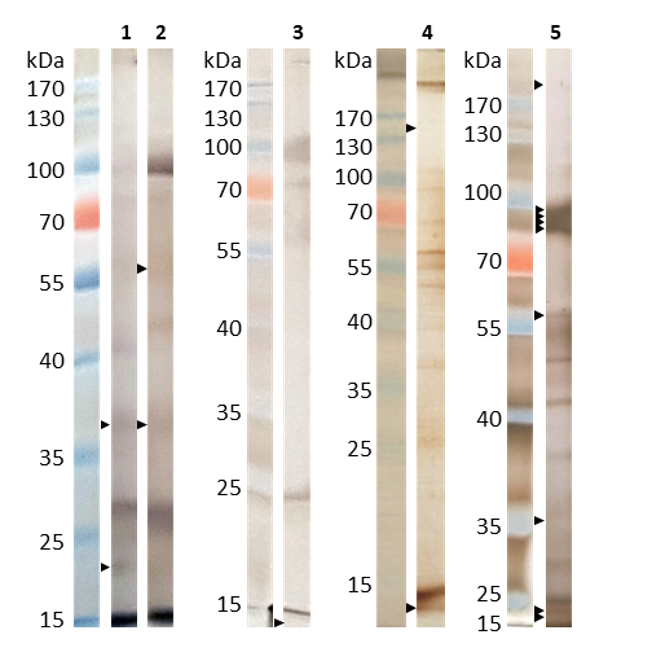

Supplement: Supplementary Figure 4 — Band pattern of pulled-down eluates with different monoclonal antibodies. SDS-PAGEs optimised for glycoproteins followed by silver staining (SilverQuest™, Invitrogen) were performed with eluates from antibody pull-down approaches. Arrows indicate the expected/predicted molecular size based on the amino acid sequence (without posttranslational modification like glycosylation) of most significant and abundant proteins identified (Table 1 and Supplementary Table 1). Lane 1: Fh-mumab-LEA_1; Lane 2: Fh-mumab-LEA_2; Lane 3: Fh-mumab-LEA_3; Lane 4: Fh-mumab-LEA_4; Lane 5: Fh-mumab-PNA_1. Common bands from co-eluted IgM-antibodies are Ig-light and heavy chains (approx. 27 and 72 kDa), J-chain (approx. 15 kDa), a precipitate of J- and light chain (approx. 42–45 kDa), and partially reduced IgM-immunoglobulins (smear around 100 kDa). A pre-stained protein ladder (PageRuler 26616, Thermo Fisher) was used as molecular marker. [file Image4.tif]
